# An ultralow-cost portable centrifuge from discarded materials for medical applications

**DOI:** 10.1038/s41598-023-30327-2

**Published:** 2023-02-22

**Authors:** Jovany J. Franco, Tatsuo Nagata, Takayuki Okamoto, Shizuo Mukai

**Affiliations:** 1 Department of Ophthalmology, Harvard Medical School, Massachusetts Eye and Ear, 243 Charles St, Boston, MA 02114 USA; 2 Department of Ophthalmology, University of Occupational and Environmental Health, Kitakyushu, Fukuoka Japan; 3 Nagoya University Graduate School of Medicine, Nagoya, Japan; 4 Retina Service, Massachusetts Eye and Ear, Boston, MA USA

**Keywords:** Health care economics, Drug delivery, Mechanical engineering

## Abstract

Reliable centrifugation for medical applications has historically required access to expensive, bulky, and electricity-dependent commercial devices, which are generally unavailable in resource-poor settings. Although several portable, low-cost, non-electric centrifuges have been described, these solutions have predominately been designed for diagnostic applications requiring sedimentation of relatively small volumes. Moreover, construction of these devices frequently requires access to specialized materials and tools that are often unavailable in underserved areas. Herein, we describe the design, assembly, and experimental validation of the CentREUSE—an ultralow-cost, portable, discarded material-based, human-powered centrifuge for use in therapeutic applications. The CentREUSE demonstrated a mean centrifugal force of 10.5 relative centrifugal force (RCF) ± 1.3. Sedimentation of 1.0 mL triamcinolone acetonide suspension for intravitreal use after 3 min of CentREUSE centrifugation was comparable to that achieved after 12 h of gravity-mediated sedimentation (0.41 mL ± 0.04 vs. 0.38 mL ± 0.03, *p* = 0.14). Sediment compactness after 5 min and 10 min of CentREUSE centrifugation was similar to that observed after centrifugation with a commercial device for 5 min at 10 RCF (0.31 mL ± 0.02 vs. 0.32 mL ± 0.03, *p* = 0.20) and 50 RCF (0.20 mL ± 0.02 vs. 0.19 mL ± 0.01, *p* = 0.15), respectively. Templates and instructions for construction of the CentREUSE are included as part of this open-source publication.

## Introduction

Centrifugation is a crucial step in many diagnostic tests and therapeutic interventions^1–4^. However, achieving adequate centrifugation has historically required access to expensive, bulky, and electricity-dependent commercial devices, which are generally unavailable in resource-poor settings^2,4^. In 2017, the Prakash group introduced a diminutive, hand-powered, paper-based centrifuge (termed the “paperfuge”) constructed from readily available materials at a cost of 0.20 United States dollars ($)^2^. The paperfuge has since been deployed in resource-poor settings for small-volume diagnostic applications (e.g., density-based separation of blood components in capillary tubes for detection of malaria parasites), thus demonstrating the real-world utility of an ultralow-cost, portable, human-powered centrifuge^2^. Since then, several other compact, low-cost, non-electric centrifugation devices have been described^4–10^. However, most of these solutions—much like the paperfuge—have been designed for use in diagnostic applications requiring sedimentation of relatively small volumes, and thus cannot be used for centrifugation of larger samples. Moreover, assembly of these solutions frequently requires access to specialized materials and tools that are often unavailable in underserved areas^4–10^.

Herein, we describe the design, assembly, and experimental validation of a centrifuge constructed from commonly discarded materials, termed the CentREUSE, that builds on the paperfuge to allow for use in therapeutic applications—situations that commonly require sedimentation of larger volumes^1,3^. As proof of concept, we test the device by means of a real-world ophthalmic intervention: sedimentation of triamcinolone acetonide (TA) suspension for subsequent injection of pelleted drug into the vitreous of the eye. Although centrifuge-concentrated TA is an established low-cost intervention employed for long-term management of several ocular conditions, the need for a commercial centrifuge during drug preparation represents a significant barrier to use of the therapy in resource-poor settings^1–3^. Sedimentation results achieved with our centrifuge are compared to those produced by conventional commercial centrifugation. Templates and instructions for construction of the CentREUSE are included as part of this open-source publication under “Supplementary Information.”

## Methods

### CentREUSE device assembly

The CentREUSE can be constructed almost entirely from discarded materials. Two copies of the semicircular template (Supplemental Fig. S1) are each printed on standard U.S. letter copy paper (215.9 mm × 279.4 mm). When adjoined, the two semicircular templates outline three key structural features of the CentREUSE device, including (1) the outer margin of the 247 mm rotating disc, (2) outlines for two slots designed to accommodate 1.0 mL syringes (with cap and amputated plunger stem), and (3) two markers indicating sites for perforations to allow passing of string through the disc.

The templates are adhered (e.g., using multipurpose glue or adhesive tape) to corrugated cardboard (minimum dimensions: 247 mm × 247 mm) (Supplemental Fig. S2a). Standard 'A' flute cardboard (4.8 mm thickness) was utilized in this study, although corrugated cardboard of similar thickness—such as from discarded shipping boxes—can be used. Using a sharp instrument (e.g., razor blade or scissors), the cardboard is cut along the outer disc margin delineated on the template (Supplemental Fig. S2b). Next, using a narrow, sharp instrument (e.g., ballpoint pen tip), two full-thickness perforations are created at a radius of 8.5 mm, in accordance with the markers depicted on the template (Supplemental Fig. S2c). The two slots for 1.0 mL syringes are then created using a sharp-tipped instrument (e.g., razor blade) to carve out the slots from the template and underlying cardboard face layer; care should be taken not to violate the corrugated layer or remaining face layer underneath (Supplemental Fig. S2d,e). Next, a string (e.g., 3 mm cotton cooking twine, or any string with similar thickness and tensile properties) is passed through the two perforations and tied to make a loop, aiming for a length of approximately 30 cm on each side of the disc (Supplemental Fig. S2f).

**Figure 1:**
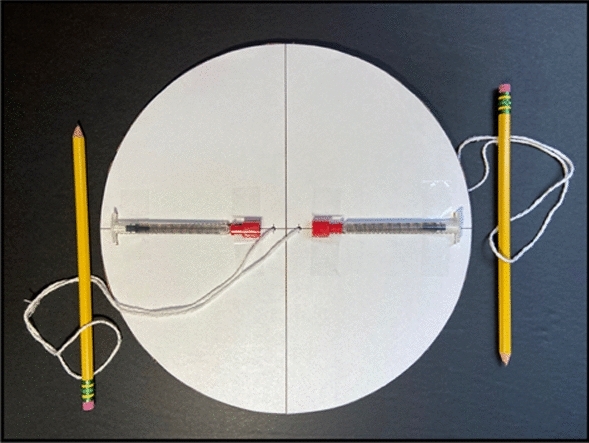
Assembled CentREUSE device.

Two 1.0 mL syringes are filled to approximately equal volumes (e.g., 1.0 mL of TA suspension) and capped. Next, the syringe plunger stem is cut at the level of the barrel flange (Supplemental Fig. S2g,h). The barrel flange is then covered with a layer of adhesive tape to prevent ejection of the amputated plunger during device use. Each 1.0 mL syringe is then placed within a syringe slot with the cap directed toward the center of the disc (Supplemental Fig. S2i). Each syringe is then adhered to the disc at a minimum of two points using tape (Supplemental Fig. S2j). Finally, two handles (e.g., pencils or similarly sturdy, stick-shaped instruments) are placed at each end of the string within the loop, thus completing centrifuge assembly (Fig. 1).

### CentREUSE device use

Instructions for use of the CentREUSE device parallel those for traditional whirligig toys. Rotation is initiated by holding one handle in each hand. With strings slightly lax, the disc is swung either forward or backward to induce forward or backward rotation of the disc, respectively. This is performed several times in a slow, controlled manner to cause coiling of the strings. This motion is then halted. As the strings begin to uncoil, the handles are pulled firmly until the strings are taut, inducing rotation of the disc. Once the strings fully uncoil and begin to re-coil, the handles should be relaxed slowly. As the string begins to uncoil again, the same series of motions are applied to maintain rotation of the device (Video S1).

For applications requiring centrifugation-assisted sedimentation of a suspension, rotation of the device is sustained until satisfactory pelleting is achieved (Supplemental Fig. S3a,b). The compound pellet will build at the plunger end of the syringe barrel, with the supernatant concentrated toward the tip of the syringe. The supernatant is then expelled by removing the tape covering the barrel flange and introducing a second plunger to slowly push the native plunger toward the tip of the syringe, halting when the compound pellet is reached (Supplemental Fig. S3c,d).

### Calculation of CentREUSE rotational speed and relative centrifugal force

**Figure 2:**
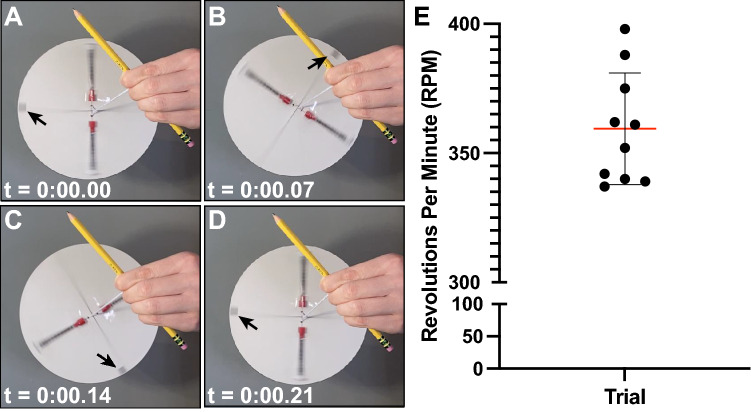
Quantification of rotational speed achieved with CentREUSE device. (**A**–**D**) Succession of representative images depicting time (min:sec.ms) transpired for completion of a full device rotation. Arrow denotes tracking marker. (**E**) Quantification of RPMs achieved with the CentREUSE. Lines represent mean (red) ± SD (black). Points represent individual 1 min trials (*n* = *10*).

To derive rotational speed, a CentREUSE device loaded with two water-filled 1.0 mL syringes was recorded using a high-speed camera (240 frames per second) for 1 min after achieving a steady oscillatory state. A marker near the edge of the rotating disc was tracked manually using frame-by-frame analysis of the recording to derive revolutions per minute (RPM) (Fig. 2a–d). This was repeated for *n* = *10* trials. Relative centrifugal force (RCF) at the midpoint of the syringe barrel was then calculated utilizing the following formula:

$$ RCF = 1.118 \times 10^{ - 5} \times r \times RPM^{2} $$

The midpoint of the syringe barrel was assumed to lie at a radius of rotation of *r* = 72.25 mm.

### Comparison of CentREUSE centrifugation to alternative techniques for TA sedimentation

1.0 mL syringes containing TA injectable suspension (40 mg/mL, Amneal Pharmaceuticals, Bridgewater, New Jersey, USA) were centrifuged for 3, 5, and 10 min using the CentREUSE. Sedimentation with this technique was compared to that achieved after centrifugation for 5 min using an Eppendorf 5810R table-top centrifuge (Hamburg, Germany) with an A-4-62 rotor at 10, 20, and 50 RCF. Sedimentation was also compared to that achieved with gravity-dependent sedimentation at multiple timepoints from 0 to 720 min. A total of *n* = *9* independent replicates were performed for each procedure.

### Statistical analysis

All statistical analyses were performed in Prism 9.0 software (GraphPad, San Diego, U.S.A.). Values reported as mean ± standard deviation (SD) unless otherwise stated. Group means were compared using two-tailed *t*-tests with Welch’s correction. Alpha was defined as 0.05. For gravity-dependent sedimentation, a one-phase exponential decay model was fitted via least squares regression, with replicate *y* values for a given *x* value considered as an individual point.

$$y={(y}_{o}-plateau)*{e}^{-K*x}+plateau$$

where, *x* is time in minutes. *y* is sediment volume. *y*_*0*_ is the *y* value when *x* is zero. *Plateau* is the *y* value at infinite minutes. *K* is the rate constant, expressed in inverse minutes.

### Ethics statement

This study was deemed exempt from review by the Institutional Review Board.

## Results

The CentREUSE device demonstrated reliable, controlled, non-linear oscillation when loaded with two standard 1.0 mL syringes, each filled with 1.0 mL of water (Video S1). Across *n* = *10* trials (1 min each), the CentREUSE demonstrated a mean rotational speed of 359.4 RPM ± 21.63 (range = 337–398), thus generating a calculated mean centrifugal force of 10.5 RCF ± 1.3 (range = 9.2–12.8) (Fig. 2a–e).

**Figure 3:**
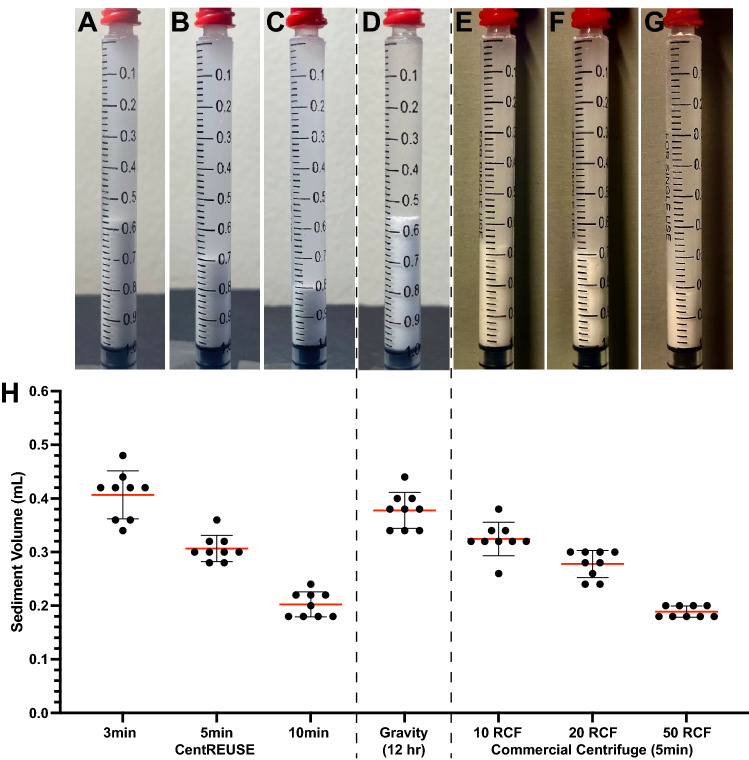
Comparison of TA sediment compactness achieved with CentREUSE centrifugation vs. gravity-mediated sedimentation vs. standard commercial centrifugation (**A**–**C**). Representative images of sedimented TA suspension in a 1.0 mL syringe after 3 min (**A**), 5 min (**B**), and 10 min (**C**) of CentREUSE use. (**D**) Representative image of sedimented TA after 12 h of gravity-mediated sedimentation. (**E**–**G**) Representative images of sedimented TA after 5 min of standard commercial centrifugation at 10 RCF (**E**), 20 RCF (**F**), and 50 RCF (**G**). (**H**) Quantification of sediment volumes achieved with the CentREUSE (3, 5, and 10 min), gravity-mediated sedimentation (12 h), and standard commercial centrifugation after 5 min (at 10, 20, and 50 RCF). Lines represent mean (red) ± SD (black). Points represent independent replicates (*n* = *9* for each condition).

Several techniques for sedimentation of TA suspension in 1.0 mL syringes were assessed and compared to CentREUSE centrifugation. After 12 h of gravity-dependent sedimentation, a sediment volume of 0.38 mL ± 0.03 was achieved (Supplemental Fig. S4a,b). Gravity-dependent sedimentation of TA was fitted to a one-phase exponential decay model (adjusted R^2^ = 0.8582), yielding a calculated plateau of 0.3804 mL (95% confidence interval: 0.3578 to 0.4025) (Supplemental Fig. S4c). The mean sediment volume of 0.41 mL ± 0.04 produced by the CentREUSE after 3 min was similar to the mean of 0.38 mL ± 0.03 observed with gravity-dependent sedimentation after 12 h (*p* = 0.14) (Fig. 3a,d,h). CentREUSE use for 5 min produced a significantly more compact volume of 0.31 mL ± 0.02, compared to the mean of 0.38 mL ± 0.03 observed with gravity-dependent sedimentation after 12 h (*p* = 0.0001) (Fig. 3b,d,h).

The mean volume of 0.31 mL ± 0.02 produced by the CentREUSE after 5 min was similar to the mean of 0.32 mL ± 0.03 observed with a standard commercial centrifuge at 10 RCF for 5 min (*p* = 0.20) and slightly less compact than the mean of 0.28 mL ± 0.03 observed with 20 RCF for 5 min (*p* = 0.03) (Fig. 3b,e,f,h). The mean volume of 0.20 mL ± 0.02 produced by the CentREUSE after 10 min was similarly compact when compared to the mean of 0.19 mL ± 0.01 observed with a commercial centrifuge at 50 RCF for 5 min (*p* = 0.15) (Fig. 3c,g,h).

## Discussion

Herein, we describe the design, assembly, and experimental validation of an ultralow-cost, portable, human-powered, paper material-based centrifuge constructed from commonly discarded materials for use in therapeutic applications. The design, in large part, builds on the paper material-based centrifuge introduced by the Prakash group for diagnostic applications (termed the “paperfuge”) in 2017^2^. Given that centrifugation has historically required access to expensive, bulky, and electricity-dependent commercial devices, the Prakash paperfuge represented an elegant solution to the issue of unreliable access to centrifugation in resource-poor settings^2,4^. The paperfuge has since demonstrated real-world utility for several small volume diagnostic applications (e.g., density-based blood-component separation for malaria detection)^2^. Yet, to our knowledge, similar ultralow-cost, paper material-based centrifugation devices have yet to be employed in therapeutic applications—situations that often require sedimentation of larger volumes.

Given this, the purpose behind the CentREUSE is to expand the utility of paper material-based centrifugation for use in therapeutic interventions. This is achieved through several modifications to the Prakash paperfuge design. Notably, to allow for the added length of two standard 1.0 mL syringes, the CentREUSE incorporates a larger disc (radius = 123.5 mm) than that of the largest tested Prakash paperfuge model (radius = 85 mm)^2^. Moreover, to accommodate the added weight of fluid-filled 1.0 mL syringes, the CentREUSE utilizes corrugated cardboard rather than cardstock. Together, these modifications allow for centrifugation of larger volumes than those trialed in the Prakash paperfuge (i.e., two 1.0 mL syringes versus capillary tubes), while still relying on similar components: string and paper-based materials. Notably, several other human-powered, low-cost centrifuges have been described for diagnostic applications^4–10^. These include devices employing fidget spinners, salad spinners, eggbeaters, and hand-cranked torch lights to deliver rotation^5–9^. Nevertheless, most of these devices are not designed to accommodate volumes as high as 1.0 mL and are composed of materials that are typically costlier and less readily available than those used in paper material-based centrifuges^2,4–10^. Indeed, discarded paper-based materials are generally widely available; for instance, in the U.S., paper and paperboard comprise more than 20% of solid municipal waste, presenting an abundant, low- to no-cost source for construction of paper material-based centrifuges like the CentREUSE^11^. Moreover, in contrast to several other published low-cost solutions, the CentREUSE does not necessitate use of specialized equipment (e.g., three-dimensional printing hardware and software, laser cutting hardware and software, etc*.*) for construction—making the device more accessible to those in resource-low settings^4,8–10^.

As evidence of the real-world utility of our paper material-based centrifuge for therapeutic applications, we demonstrate rapid and reliable sedimentation of triamcinolone acetonide (TA) suspension for injection of pelleted drug into the vitreous of the eye—an established low-cost intervention for long-term management of various ocular conditions^1,3^. Sedimentation results after 3 min of CentREUSE use were comparable to those achieved after 12 h of gravity-mediated sedimentation. Moreover, results after 5 min and 10 min of CentREUSE centrifugation exceeded those achievable with gravity and were similar to those observed after 5 min of commercial centrifugation at 10 and 50 RCF, respectively. Notably, it was our experience that the CentREUSE produced a more clearly defined and horizontal sediment-supernatant interface when compared to that produced by the other methods tested; this is preferable since it allows for more accurate estimation of injected drug dose and more facile removal of the supernatant with minimal pellet volume loss.

The choice of this application as proof of concept is inspired by the ongoing need for improved access to long-term intravitreal steroids in resource-poor settings. Intravitreal steroids are used widely for management of several ocular disorders, including diabetic macular edema, age-related macular degeneration, retinal vascular occlusion, uveitis, radiation retinopathy, and cystoid macular edema^3,12^. Of steroids available for injection into the vitreous, TA remains the most commonly used worldwide^12^. Although preservative-free TA (PF-TA) formulations are available (e.g.,* Triesence* [40 mg/mL, Alcon, Fort Worth, USA]), benzyl-alcohol-preserved formulations (e.g.,* Kenalog-40* [40 mg/mL, Bristol-Myers Squibb, New York City, USA]) remain most popular^3,12^. Of note, the latter group of formulations are only approved by the United States Food and Drug Administration (FDA) for intramuscular and intraarticular use; thus, intraocular administration is considered off-label^3,12^. Although injected dose of intravitreal TA varies with indication and technique, the most commonly reported dose is 4.0 mg (i.e., injection volume of 0.1 mL from a solution of 40 mg/mL), typically yielding a therapeutic effect lasting approximately 3 months^1,12–15^.

In an attempt to prolong the effect of intravitreal steroids for use in chronic, severe, or recurrent ocular conditions, several long-term implantable or injectable steroid devices have been introduced including the dexamethasone 0.7 mg (Ozurdex, Allergan, Dublin, Ireland), fluocinolone acetonide 0.59 mg (Retisert, Bausch and Lomb, Laval, Canada), and fluocinolone acetonide 0.19 mg (Iluvien, Alimera Sciences, Alpharetta, Georgia, U.S.A.) devices^3,12^. Nevertheless, these devices present several potential drawbacks. In the U.S., each device is only approved for a small number of indications, limiting insurance reimbursement. Moreover, certain devices require surgical implantation and can produce unique complications, such as device migration to the anterior chamber^3,12^. Additionally, these devices are generally less readily available and significantly costlier compared to TA^3,12^; at current U.S. pricing, *Kenalog-40* costs approximately $20 per 1.0 mL suspension, while the Ozurdex, Retisert, and Iluvien implants cost approximately $1400, $20,000, and $9200, respectively. Together, these factors limit the accessibility of these devices to those in resource-poor settings.

Owing to its generally lower cost, more generous reimbursement, and greater availability, attempts have been made to prolong the effects of intravitreal TA^1,3,16,17^. Given its low solubility in water, TA remains as a depot within the eye, producing gradual and relatively constant diffusion of the drug; thus, if a larger depot is injected, the longer the effect is expected to persist^1,3^. To this end, several methods have been devised to concentrate TA suspensions prior to injection into the vitreous. While techniques relying on passive (i.e., gravity-dependent) sedimentation or Millipore filtration have been described, these approaches are relatively time-intensive and yield variable results^15–17^. In contrast, prior studies have demonstrated rapid and reliable concentration of TA (with a resulting prolongation of drug effect) through centrifugation-assisted sedimentation^1,3^. Together, the convenience, low cost, duration, and effectiveness of centrifugation-concentrated TA make the intervention an appealing option for patients in resource-low settings. Nevertheless, lack of access to reliable centrifugation may represent a significant barrier to delivery of this intervention; by ameliorating this issue, the CentREUSE may help improve the accessibility of long-term steroid treatment for patients in resource-poor settings.

## Limitations

Our study carries several limitations, including some that stem from features inherent to the CentREUSE device. The device represents a non-linear, non-conservative oscillator that relies on energy introduced by a human, thus making it impossible to deliver a precise, consistent rotational speed during use; rotational speed is subject to influence from multiple variables, such as user proficiency with the device, specific materials used for device assembly, and mass of compounds being centrifuged. This differs from commercial devices, where rotational speeds can be applied in a consistent and precise manner. Moreover, the speeds achieved by the CentREUSE may be viewed as relatively modest when compared to those achieved by other centrifugation devices^2^. Fortunately, the speeds (and associated centrifugal forces) produced by our device are sufficient for the proof-of-concept application detailed in our study (i.e., sedimentation of TA). Rotational speed could be improved by reducing the mass of the central disc^2^; this could be achieved by use of a lighter material (e.g., thinner cardboard) as long as the material is sturdy enough to accommodate two fluid-filled syringes adhered to its surface. In our case, the decision to use standard 'A' flute cardboard (4.8 mm thickness) was intentional, given that the material is commonly used in shipping boxes and thus can be readily found as discarded material. Rotational speed could also be increased by decreasing the radius of the central disc^2^. However, the radius of our platform was intentionally kept relatively large to accommodate the length of 1.0 mL syringes. If a user has interest in centrifuging a container of lesser length, then the radius can be made smaller—a change that would foreseeably yield higher rotational speeds (and potentially greater centrifugal forces).

Furthermore, we did not rigorously evaluate the impact of operator fatigue on device function. Anecdotally, several members of our group were able to use the device for 15 min without significant fatigue. A potential way of addressing operator fatigue when more prolonged centrifugation is necessitated is alternating between two or more users, if available. Additionally, we did not rigorously evaluate the durability of our device, in part, because the components of the device (i.e., cardboard and string) can easily be replaced at little or no cost if they were to become worn or damaged. Anecdotally, we used a single device for a total of more than 200 min during our experimental validation. At the end of this period, the only obvious, albeit minimal, sign of wear was along the perforations through which the strings are threaded.

Another limitation of our study is that we did not specifically measure the mass or density of sedimented TA achievable with the CentREUSE device and other techniques; instead, our experimental validation of the device is based on measurement of the compactness of sediment (in mL) as a proxy measure for density. Additionally, we did not test CentREUSE-concentrated TA in patients; nevertheless, since the TA pellets created by our device are similar to those produced using commercial centrifuges, we postulate that the effectiveness and safety of CentREUSE-concentrated TA would be similar to what has been previously reported in the literature using conventional centrifugation devices^1,3^. Additional studies quantifying actual injected TA amounts after CentREUSE-concentration may serve to further assess the real-world utility of our device for this application.

## Conclusions

To our knowledge, the CentREUSE—a device that can be easily constructed from readily available, discarded materials—is the first human-powered, portable, ultralow-cost, paper material-based centrifuge to be applied in a therapeutic setting. In addition to allowing for the centrifugation of relatively large volumes compared to other published low-cost centrifuges, the CentREUSE does not necessitate access to specialized materials and tools for construction. The demonstrated effectiveness of the CentREUSE for rapid, reliable sedimentation of TA may serve to improve accessibility of long-term intravitreal steroids for those in resource-poor settings, and thus potentially aid in the management of a wide range of ocular conditions. Moreover, the benefit of our human-powered, portable centrifuge could foreseeably extend to resource-rich settings, such as large, tertiary- and quaternary-care centers in developed nations. In these settings, availability of centrifugation devices may still be limited to clinical and research laboratories, presenting a risk for syringe contamination by human body fluids, animal products, and other hazardous substances. Moreover, these laboratories are often removed from sites of patient care delivery. This, in turn, may present a logistical barrier to providers in need of quick access to centrifugation; deployment of the CentREUSE may serve as a practical way to prepare therapeutic interventions in short order without significant interruption to patient care delivery.

Thus, in an effort to make preparation of therapeutic interventions requiring centrifugation more accessible to all, templates and instructions for construction of the CentREUSE are included as part of this open-source publication under “Supplementary Information.” We invite readers to make modifications to the CentREUSE design as needed.

## Supplementary Information

Supplementary Information 1.

Supplementary Video 1.

## Data Availability

The data that support the findings of this study are available from the corresponding author, SM, upon reasonable request.
