# Supplementary material for: An ultralow-cost portable centrifuge from discarded materials for medical applications
Source: Sci Rep. 2023 Feb 22;13:3081. doi: 10.1038/s41598-023-30327-2 (PMC9946952; doi:10.1038/s41598-023-30327-2)
Supplement: Supplementary file 1 — Supplementary Information 1. [file 41598_2023_30327_MOESM1_ESM.pdf]

SUPPLEMENTARY MATERIAL:

An Ultralow-Cost Portable Centrifuge from Discarded Materials for Medical Applications

Jovany J. Franco B.S., Tatsuo Nagata M.D., Ph.D., Takayuki Okamoto M.D.,

Shizuo Mukai M.D.

Video 1. CentREUSE device in use.

Link: https://youtu.be/PU9yHDysOiY

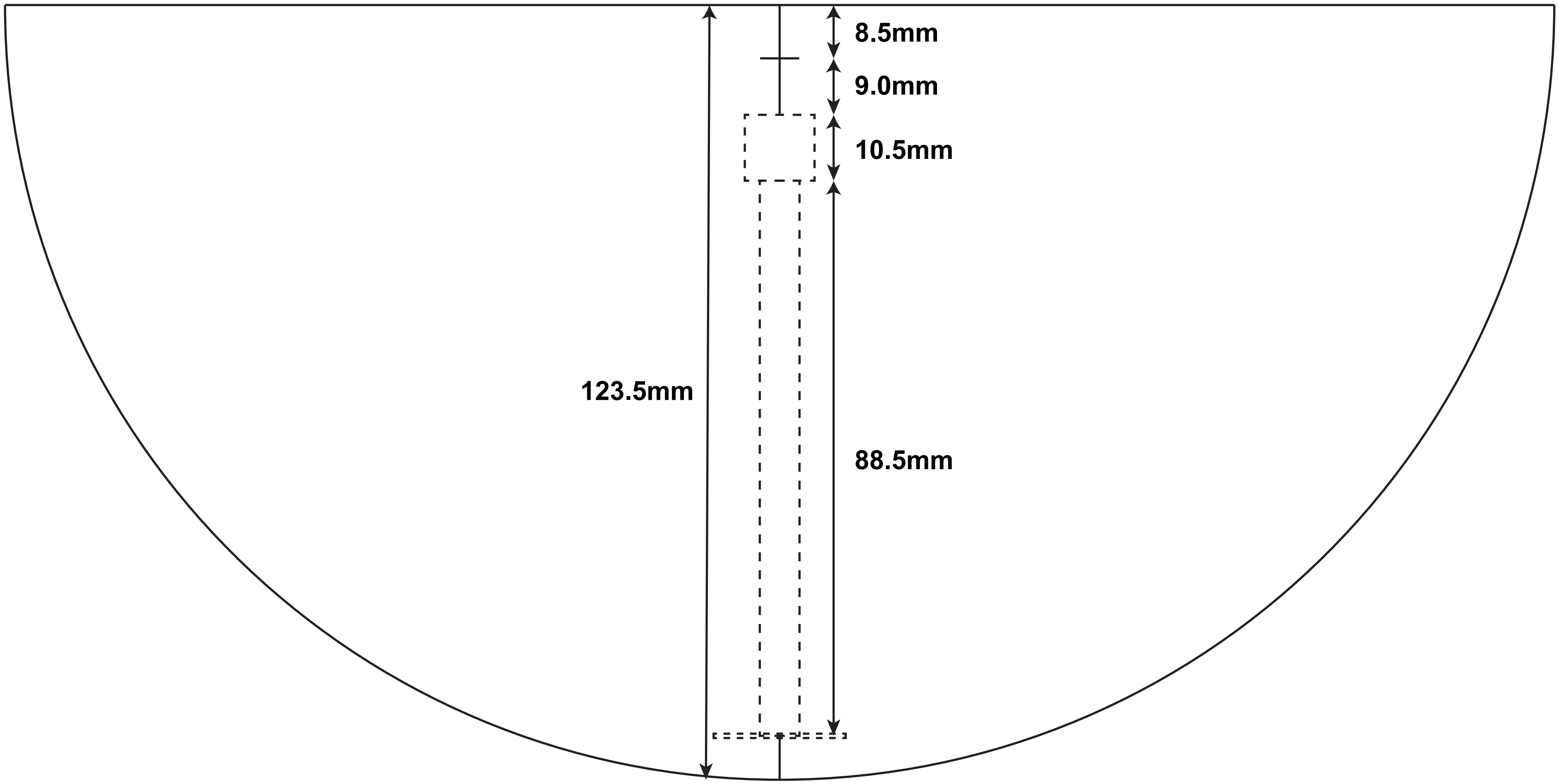

Supplemental Figure S1. Template for CentREUSE device.

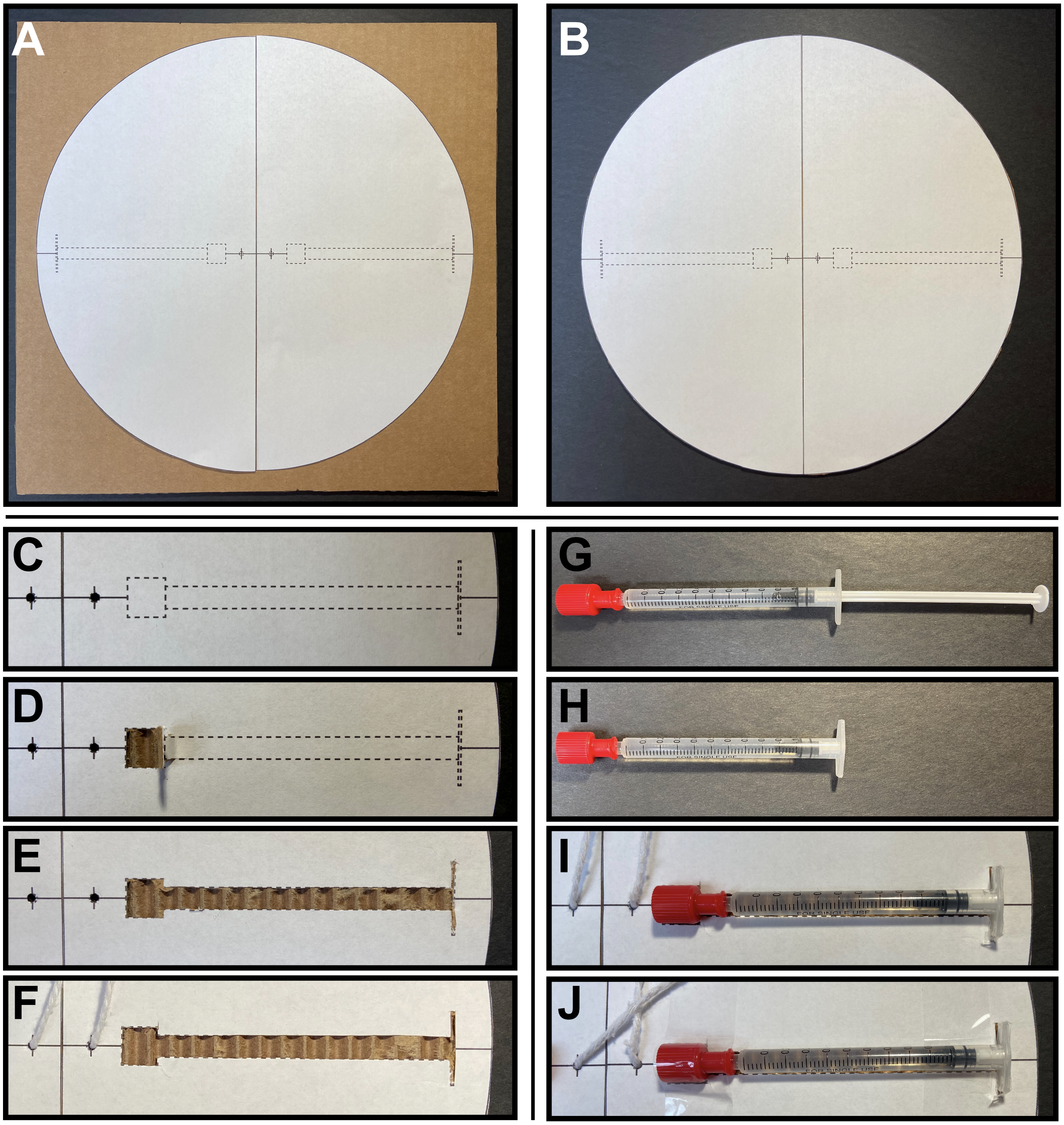

Supplemental Figure S2. Assembly of CentREUSE device. A. Two templates are adhered to corrugated cardboard. **B.** The cardboard is cut along the outer disc margin delineated on the template. **C.** Two full-thickness perforations are created at a radius of 8.5 mm. **D, E.** Two partial-thickness slots for 1.0 mL syringes are created. **F.** String is passed through the two perforations, yielding 30 cm of length on each end of the disc. **G, H.** The plunger stems of two fluid-filled 1.0

mL syringes are amputated at the level of the barrel flange. **I, J.** Each 1.0 mL syringe is placed within a syringe slot with the cap directed toward the center of the disc (**I**) and subsequently adhered to the disc (**J**).

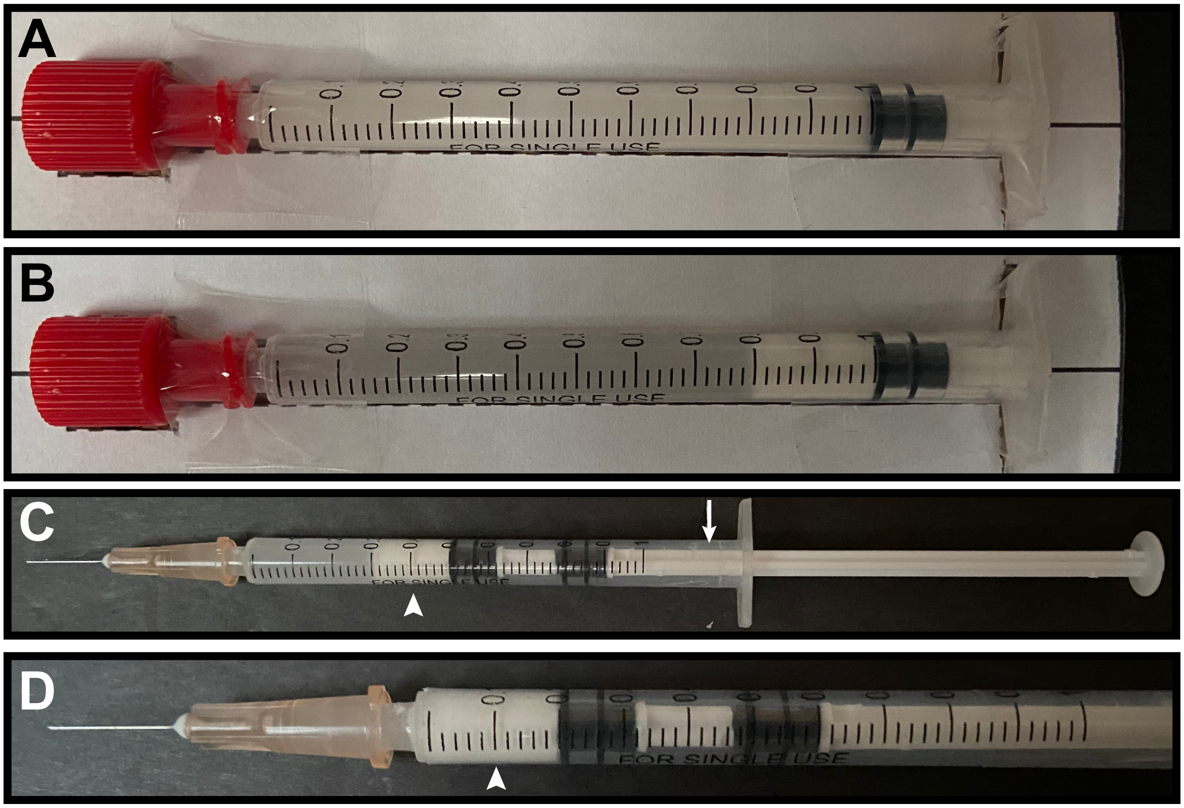

Supplemental Figure S3. Procedure for CentREUSE-mediated sedimentation of TA. A, B. TA injectable suspension **(A)** is centrifuged using the CentREUSE device until desired degree of sedimentation is achieved **(B)**. **C, D.** A second plunger (arrow) is introduced in the flange-end of the barrel **(C)** to slowly push the native plunger toward the tip of the syringe, removing the supernatant and preserving the TA pellet (arrowhead) **(D)**.

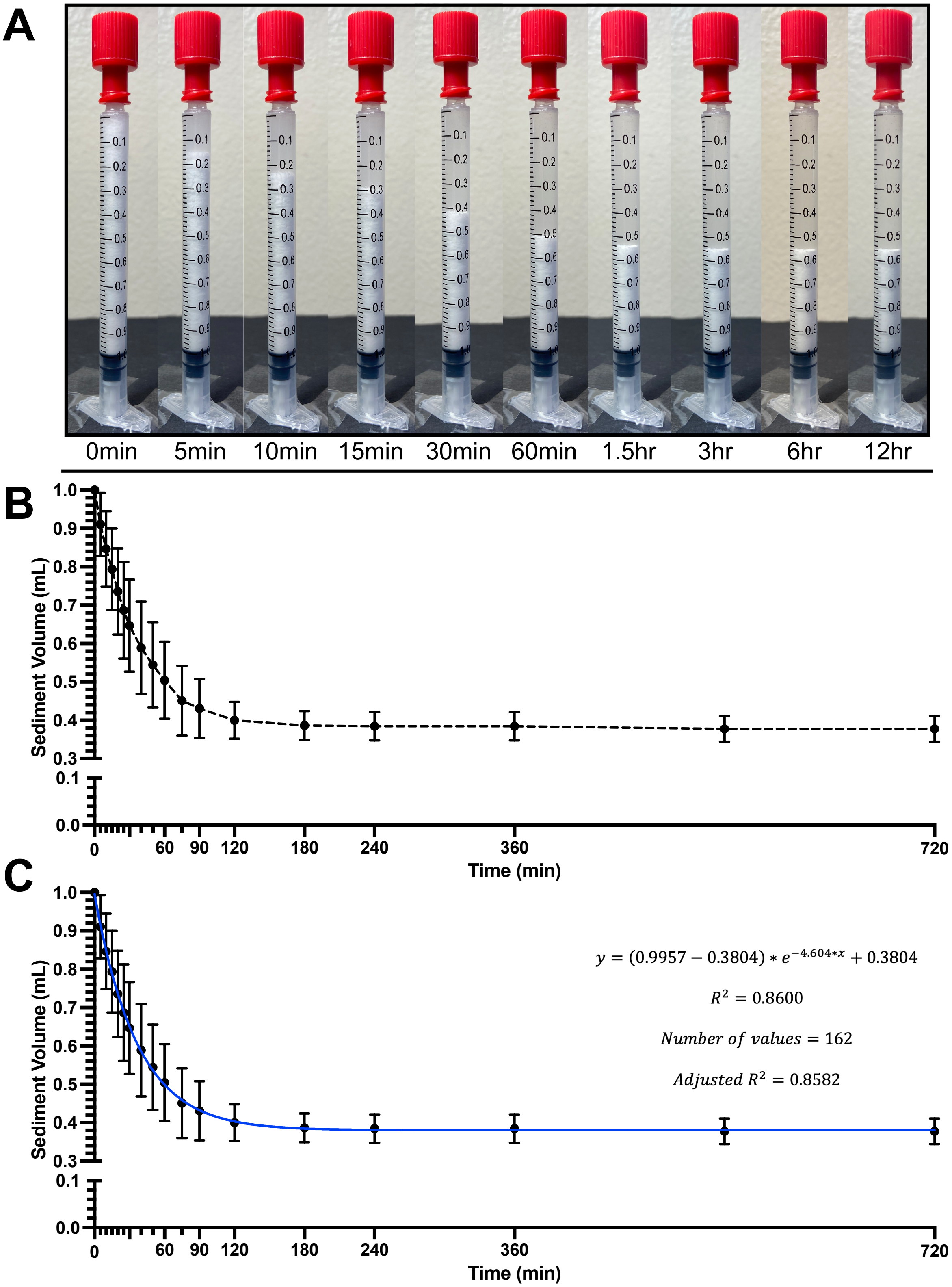

Supplemental Figure S4. Time-course of gravity-mediated sedimentation of TA. A. Succession of representative images depicting gravity-mediated sedimentation of TA at multiple

timepoints over 12 hrs. **B.** Quantification of sediment volumes achieved with gravity-mediated sedimentation at multiple timepoints over 12 hrs. Points represent mean sediment volume at a given timepoint (*n=9 independent replicates*). Error bars represent ± SD. **C.** Graphical overlay (blue) and parameters for one-phase exponential decay model of gravity-mediated sedimentation.
